# Profiling of *Saccharomyces cerevisiae* transcription factors for engineering the resistance of yeast to lignocellulose-derived inhibitors in biomass conversion

**DOI:** 10.1186/s12934-017-0811-9

**Published:** 2017-11-14

**Authors:** Guochao Wu, Zixiang Xu, Leif J. Jönsson

**Affiliations:** 1 Department of Chemistry, Umeå University, 901 87 Umeå, Sweden; 2 Tianjin Institute of Industrial Biotechnology, Chinese Academy of Sciences, Tianjin, 300308 China

**Keywords:** Lignocellulosic biomass conversion, *Saccharomyces cerevisiae*, Multidrug resistance, Transcription factors, *STB5*, *YAP1*

## Abstract

**Background:**

Yeast transcription factors (TFs) involved in the regulation of multidrug resistance (MDR) were investigated in experiments with deletion mutants, transformants overexpressing synthetic genes encoding TFs, and toxic concentrations of lignocellulose-derived substances added to cultures as complex mixtures or as specific compounds, viz. coniferyl aldehyde, 5-hydroxymethylfurfural, and furfural.

**Results:**

In the presence of complex mixtures of toxic substances from spruce wood, transformants overexpressing *YAP1* and *STB5*, TFs involved in oxidative stress response, exhibited enhanced relative growth rates amounting to 4.589 ± 0.261 and 1.455 ± 0.185, respectively. Other TFs identified as important for resistance included *DAL81*, *GZF3*, *LEU3*, *PUT3*, and *WAR1*. Potential overlapping functions of *YAP1* and *STB5* were investigated in experiments with permutations of deletions and overexpression of the two genes. *YAP1* complemented *STB5* with respect to resistance to 5-hydroxymethylfurfural, but had a distinct role with regard to resistance to coniferyl aldehyde as deletion of *YAP1* rendered the cell incapable of resisting coniferyl aldehyde even if *STB5* was overexpressed.

**Conclusions:**

We have investigated 30 deletion mutants and eight transformants overexpressing MDR transcription factors with regard to the roles the transcription factors play in the resistance to toxic concentrations of lignocellulose-derived substances. This work provides an overview of the involvement of thirty transcription factors in the resistance to lignocellulose-derived substances, shows distinct and complementary roles played by *YAP1* and *STB5*, and offers directions for the engineering of robust yeast strains for fermentation processes based on lignocellulosic feedstocks.
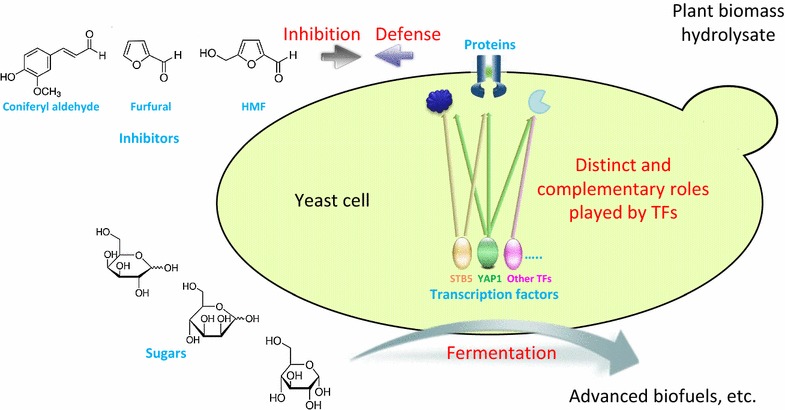

**Electronic supplementary material:**

The online version of this article (10.1186/s12934-017-0811-9) contains supplementary material, which is available to authorized users.

## Background

Lignocellulosic biomass is an abundant renewable resource for production of advanced biofuels and other products that can be obtained through microbial fermentation processes. Prior to saccharification and fermentation, the lignocellulose is typically pretreated to make the cellulose more accessible to cellulolytic enzymes. During pretreatment a wide range of fermentation inhibitors are generated together with the fermentable sugars [1]. The fermentation inhibitors, which include phenolic compounds, furan aldehydes, and aliphatic acids, are toxic to yeast cells and can cause various stress conditions during the ethanolic fermentation. Coniferyl aldehyde, furfural and HMF (5-hydroxymethyl-2-furaldehyde) are examples of lignocellulose-derived inhibitors the effects of which have been much studied in model experiments. The response to inhibitors includes transcriptional reprogramming of gene expression to provide a proteome that is better suited to cope with the induced stress.

Multidrug resistance (MDR) is the ability to acquire simultaneous resistance to distinct drugs or chemicals of a wide variety of structures and function. *Saccharomyces cerevisiae* has been extensively studied as a model organism with regard to MDR [2]. In yeast, MDR membrane proteins are divided into two superfamilies: the ATP-binding cassette (ABC) superfamily and the major facilitator superfamily (MFS). They are regulated by various transcription factors (TFs) and are responsible for yeast cell survival under many kinds of stress conditions [3, 4].

TFs bind to unique DNA elements and cause either transcriptional activation or repression. Many transcriptional activators and repressors are involved in the regulation of the expression of MDR proteins, and they are classified into different families due to their structural or functional similarity [5].

In our previous work, we found by deoxyribonucleic acid microarray analysis that the MDR TF Yap1p of *S. cerevisiae* was related to resistance to lignocellulose-derived inhibitors [6]. We found that the overexpression of the gene resulted in enhanced resistance to coniferyl aldehyde, HMF and spruce wood hydrolysate [7]. Yap1p is involved in MDR and is the main regulator in response to oxidative stress [8]. We have studied the mechanism of transcriptional activation by Yap1p and the target genes of the TF [9]. However, the control of gene expression in response to stress conditions is mediated by several TFs. The exact roles of the TFs and their relationships to each other during stress response are still unclear.

In this study, we selected 30 TFs that regulate MDR proteins from either the ATP-binding cassette (ABC) superfamily or the major facilitator superfamily (MFS), and investigated their involvement in the resistance to chemical stress induced by lignocellulose-derived inhibitors. The deletion mutants of the 30 TFs were screened in experiments covering simple inhibition scenarios (separate compounds including coniferyl aldehyde, furfural and HMF) and complex inhibition scenarios (using the pretreatment liquid from pretreated sugarcane bagasse and Norway spruce). The involvements of the TFs in the response to the simple inhibition scenarios, which represent partially the inhibition effects of the pretreatment liquids, were compared with that of the pretreatment liquids. Eight TFs were selected and overexpressed, and the resistance of the transformants to the two pretreatment liquids was evaluated. Furthermore, the two TFs encoded by *YAP1* and *STB5* were studied in detail in experiments with permutations of deletions and overexpression of the two genes to investigate if they have distinct or overlapping roles. This investigation elucidated the adaptation of yeast to harsh conditions prevailing during fermentation of pretreated lignocellulose to desired products, and is helpful for the design of genetically engineered yeast with improved performance in biomass conversion. This investigation is also helpful for understanding the roles of specific MDR-related transcription factors in the response to toxic compounds.

## Methods

### Microbial strains and mutants

*Escherichia coli* DH5α was used for all cloning procedures. The bacterium was grown in LB medium and cloning procedures were carried out by standard methods [10]. *S. cerevisiae* strain BY4741 (MATa; his3Δ 1; leu2Δ 0; met15Δ 0; ura3Δ 0), and deletion mutants of 30 TFs involved in MDR were obtained from EUROSCARF (Frankfurt, Germany), and were cultivated on only Synthetic Complete (SC) medium before they were exposed to the inhibitors and inhibitory substances.

### Plasmid construction

The vectors used in this study were derived from the episomal yeast shuttle vector pAJ401 [11]. The pAJ401 plasmid contains the ColE1 origin of replication, the β-lactamase gene, the 2 μ origin of replication, the *URA3* selection marker and the *PGK1* expression cassette. As a negative control, a plasmid denoted PB (plasmid backbone) was used (pAJ401 without any insert between the *PGK1* promoter and the terminator [12]). The pAJ401-derived plasmid with the *YAP1* gene was constructed previously [7]. The synthesis of seven other genes and the construction of the corresponding pAJ401 expression plasmids were performed by Gene Oracle (Santa Clara, CA, USA). The integrity of the synthesized gene and the construction was checked by DNA sequencing.

### Preparation of plasmid DNA and transformation of *S. cerevisiae*

To amplify the plasmids, competent *E. coli* DH5α cells were transformed with the plasmids by using calcium chloride and heat-shock treatment [10].

The *E. coli* transformants were selected on LB agar plates with ampicillin. Transformants from the selective agar plates were grown at 37 °C in shake flasks with 50 ml of LB medium with ampicillin. Purification of plasmids was carried out with a Plasmid Purification Kit (Qiagen).

To obtain the same strain background as in the experiments with deletion mutants, the auxotrophic *S. cerevisiae* strain BY4741 was transformed with the three plasmids (negative control plasmid denoted PB, and expression plasmids of *YAP1* and *STB5*) by electroporation. Electroporation was carried out as previously described [7]. The auxotrophic *S. cerevisiae* deletion mutant of *YAP1* was transformed with the *STB5* overexpression plasmid (the resulting transformant was named SIY), and the deletion mutant of *STB5* was transformed with the *YAP1* overexpression plasmid (the resulting transformant was named YIS), using the same electroporation method as described above.

Three different colonies were picked for each of the transformants and evaluated in the microtitre plate experiments and flask experiments.

### Inhibitory substances

Three model inhibitory compounds, one phenolic compound (coniferyl aldehyde) and two furan aldehydes (furfural and HMF), were selected for studies of the effects of stress caused by specific compounds. Coniferyl aldehyde, furfural and HMF were obtained from Sigma-Aldrich.

The liquid phases from two types of lignocellulosic biomass pretreated at high temperature and under acidic conditions were used for studies of stress caused by complex mixtures of inhibitory compounds. The lignocellulosic biomass used was Norway spruce and sugarcane bagasse. The spruce pretreatment liquid used in the experiments was obtained from a slurry from the Swedish Biorefinery Demo Plant (Örnsköldsvik, Sweden). Wood chips of Norway spruce (*Picea abies*) were treated in a continuous mode in a 30-l reactor at a temperature of 204 °C and with a residence time of 7–8 min. The feed rate was 24 kg/h (dry weight) and the spruce was impregnated with sulfur dioxide (1.2–1.3 kg/h). The pH after pretreatment was 1.4. The dry-matter content after pretreatment was 12%. The pretreated material was cooled and stored at 4 °C until further use.

The pretreatment of sugarcane bagasse was performed similarly in the same demonstration plant. The sugarcane bagasse was treated in a continuous mode in the 30-l reactor at a temperature of 188 °C and with a residence time of 10 min. The feed rate was 24 kg/h (dry weight) and the bagasse was impregnated with sulfur dioxide (0.3 kg/h). The pH after pretreatment was 2.1. The dry-matter content after pretreatment was 18%. The pretreated material was cooled and stored at 4 °C until further use.

The contents of the predominant monosaccharides, furan aldehydes, and aliphatic acids of the pretreatment liquid of the slurries of pretreated Norway spruce and sugarcane bagasse were analyzed by MoRe Research (Örnsköldsvik, Sweden). The spruce pretreatment liquid (SPL) contained (in g/l): arabinose, 2.7; galactose, 3.5; glucose, 18.2; mannose, 16.3; xylose 12.4; furfural, 2.4; HMF, 0.2; acetic acid, 4.8; formic acid, 1.8; levulinic acid, 0.8. The bagasse pretreatment liquid (BPL) contained (g/l): arabinose, 3.1; xylose, 34.6; galactose, 1.6; glucose, 10.6; mannose, 2.4; furfural, 8.9; HMF, 1.1; acetic acid, 14.0; formic acid, 0.3; levulinic acid, 0.5.

### Experiments with microtitre plates

Five sets of experiments were carried out with the deletion mutants, and one set of experiments was carried out with the BY4741 transformants of the 8 transcription factors. Another set of experiments was for comparing *STB5* and *YAP1*, and the *YAP1* deletion mutant (M-Y), the *STB5* deletion mutant (M-S), the *YAP1* transformant of BY4741 (T-Y), the *STB5* transformant of BY4741 (T-S), the SIY transformant and the YIS transformant were included in this set of experiment, as well as the two controls (BY4741 and the BY4741 transformant carrying the plasmid backbone, PB, without insert). The resistance of the deletion mutants and the transformants was evaluated by comparing the relative growth rate, which was calculated based on cell growth (OD_620_). For each deletion mutant or transformant, all sets of experiments were performed in triplicates. For the deletion mutants, technical triplicates were used with the same mutant. For the transformants, biological triplicates were used with three different colonies picked for each transformant. The average OD values of triplicates were used in the evaluation.

#### Preparation of precultures

The deletion mutants were inoculated in 50 ml Falcon tubes containing 10 ml of SC medium (with 200 mg/l uracil). Three different colonies of each transformant were selected and inoculated in different 50 ml Falcon tubes containing 10 ml of SC-Ura medium. The Falcon tubes were incubated at 30 °C with agitation. The cultures were harvested after an overnight cultivation, and inoculated again into 50 ml Falcon tubes containing 10 ml of SC. The cultures were incubated for 4–8 h, and were harvested in the exponential growth phase by centrifugation (Eppendorf 5810R, Eppendorf AG, Hamburg, Germany) at 8000 rpm for 2 min. The cells were then resuspended in an appropriate volume of sterilized deionized water to give a start inoculum with a biomass concentration of 0.12 g/l DW (dry weight).

Hundred microliters of triple concentrated SC or SC-Ura medium and 100 ml of cell suspension were added to each well of a microtitre plate (Nunc, Roskilde, Denmark). In the experiments with the deletion mutants, 100 μl of inhibitor solution or diluted pretreatment liquid were added to the culture to a final concentration of 1.0 mM coniferyl aldehyde, 10.4 mM (1.0 g/l) furfural, and 14.0 mM HMF. In the experiments with the transformants, final concentrations of 1.2 mM coniferyl aldehyde, 17.0 mM furfural and 24.0 mM HMF were used. Since coniferyl aldehyde is hard to dissolve in pure water, it was first dissolved in a few milliliters of ethanol before the addition of water. The final concentration of the ethanol added was below 0.1% (v/v), and its effect on yeast cell growth was negligible. In the experiments with microtitre plates, 15% (v/v) spruce pretreatment liquid or 10% (v/v) bagasse pretreatment liquid were used, and the pH of the pretreatment liquids was adjusted to 6.0 before adding them into the wells of the microtiter plates. The pH of the cultures was adjusted to 6.0 at the start of the experiment, and the final pH of the cultures was measured. The final volume in each well in all the microtitre plate experiments was 300 μl out of the maximum volume 330 μl.

The cultivation was anaerobic as the oxygen was depleted shortly after the start of the cultivation. Airproof adhesive film was used to seal the microtitre plates to avoid well-to-well contamination and sample evaporation, and to keep the anaerobic environment. Control wells filled with only culture medium were included to confirm there was no cross contamination. A start OD (optical density) was measured at 620 nm (Victor^2^ 1420 Multilabel Counter, Perkin Elmer, Waltham, MA, USA). The plate was then incubated at 30 °C in a shaker incubator (Ecotron, Infors AG, Bottmingen, Switzerland) with a shaking speed of 180 rpm. The OD was measured after 12 and 24 h, and extra time points (4, 9, 18, 36, 38, 40, 42, 48 and 60 h) in some sets of the experiments.

### Fermentation in glass flasks

To investigate the resistance of the deletion mutants and transformants of *STB5* and *YAP1* towards coniferyl aldehyde and HMF more thoroughly, fermentation experiments were carried out in 100-ml glass flasks. The flasks were incubated at 30 °C in the Ecotron incubator set at 180 rpm.

Inoculum cultures of BY4741, M-Y, M-S, SIY and YIS were prepared in the same way as in the microtiter plate experiments. The cells were resuspended in a suitable amount of sterile deionized water to yield an inoculum of 0.12 g/l (DW) in all fermentation flasks. Thirty glass flasks were filled with SC medium, of which 18 contained medium with uracil (for BY4741, M-Y and M-S) and 12 medium without uracil (for SIY and YIS). The flasks were inoculated with 10 ml of cell suspension and the total volume was 30 ml. The experiment was performed in duplicates, and each of the mutants and transformants was tested without any inhibitor present in the medium (control), with 1.1 mM initial concentration of coniferyl aldehyde, and with 24.0 mM initial concentration of HMF. The pH of the cultures was adjusted to 6.0 at the start of the experiment, and the final pH of the cultures was measured.

The OD, the glucose concentration and the ethanol concentration were measured in samples taken after 0, 8, 16, 40, 64, 88, 112, and 136 h. The OD was measured at 620 nm. The glucose concentration was measured with a glucometer (ACCU-CHEK^®^ Aviva, Roche Diagnostics GmbH, Mannheim, Germany). The ethanol production was measured by using an ethanol determination kit (Ethanol UV-method, Boehringer Mannheim GmbH, Mannheim, Germany). Prior to the ethanol assay, the fermentation samples were heat-treated at 80 °C for 15 min to stop the enzymatic reactions. The cell viability was measured in samples taken after 0 and 8 h, with the Yeast Viability Kit (Molecular Probes, Invitrogen, Carlsbad, CA, USA). The cell viability was determined by counting the number of viable and dead cells in a fluorescence microscope (Axioimager Z1, Carl Zeiss MicroImaging GmbH, Göttingen, Germany). At least 200 cells of each fermentation sample were counted to determine the cell viability.

### Calculations

In the screening experiments with microtiter plates, while measuring the OD_620_ value, the samples were not always within the optimal reading range of the plate reader. Therefore, a standard curve was used to establish an equation for correction of the OD_620_ values.

A yeast cell culture was diluted to seven different concentrations, and the diluted samples were measured with the plate reader. The standard curve was made for OD_620_ value correction. Calculations were carried out by using Matlab and the polynomial fitting method. The following equation was used for corrections:

$$y = 0.02396 \times (p_{1} \times x^{4} + p_{2} \times x^{3} + p_{3} \times x^{2} + p_{4} \times x + p_{5} )$$

In this equation, *x* is the OD_620_ value obtained with the plate reader (before the correction), while *y* is the calculated value of OD_620_ after the correction. The constant 0.02396 was the OD_620_ value taken in the optimal reading range of the plate reader and used for the correction. The values of the parameters (*p*_1_–*p*_5_) were obtained after the fitting.

$$\left[ {p1, \, p2, \, p3, \, p4, \, p5} \right] = [- 7.766, \, 23.749, \, - 3.345, \, 44.811, \, - 0.025]$$

The OD_620_ values of the cultures were measured after 0, 12, and 24 h, and extra time points (4, 9, 18, 36, 38, 40, 42, 48 and 60 h) in some sets of experiments. After the corrections of the OD_620_ values as described above, fitting curves of growth curves were made for each independent culture in each well of the microtitre plate. The fitting curves were made with Piecewise Cubic Hermite Interpolation (PCHI) [13] in Matlab. For each independent culture, a piecewise cubic function (monotone function) was made between *t* (time) and *y* (the corrected OD_620_ value).

$$t\left( i \right) = f\left( {y_{i} } \right),\quad{\text{i}} = 1, \, 2, \ldots ,{\text{n}}$$

In each subinterval *I*_i_ = [*y*_i_, *y*_i+1_], *f* (*y*) was a cubic polynomial [13].

$$f(y) = t_{i} H_{1} (y) + t_{i + 1} H_{2} (y) + d_{i} H_{3} (y) + d_{i + 1} H_{4} (y)$$

In the polynomial, *d*_*j*_ = *f*′(*y*_*j*_), and *j* = *i*, *i* + 1, and the *H*_*k*_(*y*) are the usual cubic Hermite basis functions for the interval *I*_i_.

The growth rate for each culture was then calculated based on the fitting curve with the following equation:

$${\text{Growth rate }} = {\text{Cell density}}/{\text{incubation time}}$$

The cell density equals four times of the corresponding initial inoculation in the same well of the microtitre plate (the inoculated cells divided two times). The incubation time, which equals the time when a cell had divided two times, was calculated based on the fitting curve.

The relative growth rate of one mutant was calculated by comparing the growth rate of the mutant to the growth rate of BY4741 in the same set of experiment.

$${\text{Relative growth}} = {\text{growth rate of the mutant or the transformant}}/{\text{growth rate of the control strain}}$$

## Results

### Screening of deletion mutants with microtitre plate assay

**Fig. 1:**
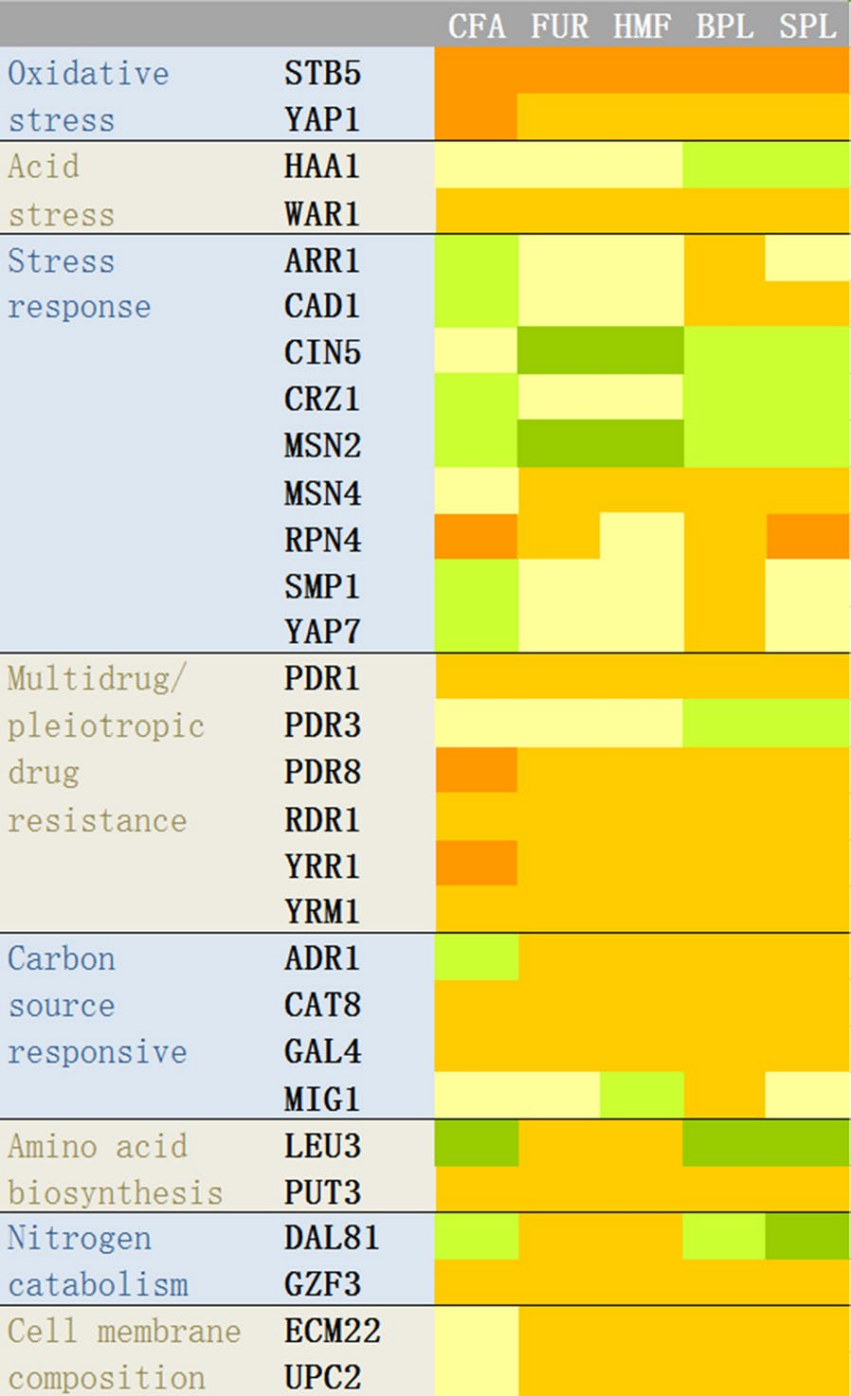
Heatmap of relative growth rate in the screening experiments with deletion mutants. The deletion mutants were cultivated with 1.0 mM coniferyl aldehyde (CFA), 10.4 mM furfural (FUR), 14.0 mM HMF, sugarcane bagasse pretreatment liquid (BPL), and spruce pretreatment liquid (SPL). The data indicate: relative growth rate < 0.5 , 0.5 ≤ relative growth rate < 0.9 , 0.9 ≤ relative growth rate ≤ 1.1 , 1.1 < relative growth rate ≤ 1.5 , and relative growth rate > 1.5 . The result with the NGG1 deletion mutant was not included in the figure, since the growth of the NGG1 deletion mutant in the SC medium was slower than that of BY4741. The numerical data for relative growth are included in Additional file 1: Table S1

Thirty deletion mutants of TFs which regulate MDR proteins of the ABC or major facilitator superfamilies were tested with a microtitre plate assay, and the relative growth rates were calculated. An overview of the result is shown in Fig. 1. Except for the NGG1 deletion mutant, all the other 29 deletion mutants displayed similar growth as the control strain BY4741 when cultured in SC-Ura medium (i.e. in the absence of inhibitors).

Looking at the whole set of mutants, there were in total 12 deletion mutants (of *STB5*, *YAP1*, *WAR1*, *PDR1*, *PDR8*, *RDR1*, *YRR1*, *YRM1*, *CAT8*, *GAL4*, *PUT3* and *GZF3*) that were more sensitive than the control to all the three specific compounds and to the two pretreatment liquids. There was one deletion mutant, that of *MSN2*, which was resistant to all the three specific compounds and to the two pretreatment liquids. The two furan aldehydes typically gave similar response in the screening experiment (for 27 out of the 29 TFs), while the response invoked by coniferyl aldehyde was frequently different from that of the two furan aldehydes (for 12 out of the 29 TFs) (Fig. 1). The two PLs gave similar response (23 out of the 29 TFs). In some cases (*CRZ1*, *LEU3*, *DAL81*), the response of the pretreatment liquids was similar to that of coniferyl aldehyde but not to that of the two furan aldehydes, while in other cases (*CIN5*, *MSN4*, *ADR1*, *ECM22*, *UPC2*), the response of the pretreatment liquids was more similar to that of the furan aldehydes than to that of coniferyl aldehyde (Fig. 1).

The relative growth rates of the deletion mutants of *STB5* and *YAP1*, which encode TFs directly related to oxidative stress, were lower than 0.9 when cultivated with the three specific inhibitors and the two pretreatment liquids (Fig. 1). In the presence of inhibitors and pretreatment liquids, the relative growth rates of the STB5 deletion mutant was always < 0.5 (Fig. 1).

The deletion mutant of *HAA1*, a transcription factor involved in acid stress response, was similar to BY4741 when cultured with coniferyl aldehyde, furfural or HMF, but was more resistant to the two pretreatment liquids. The relative growth rate of the deletion mutant of *WAR1*, another transcription factor involved in acid stress response, was < 0.9 when cultivated with all the three inhibitors and the two pretreatment liquids (Fig. 1).

The experiment suggested that the deletion mutants of *CIN5* (*YAP4*) and *MSN2*, which are two of the TFs regulating stress response of *S. cerevisiae*, were more resistant than the control to furan aldehydes and to pretreatment liquids. The two deletion mutants seem to have particularly high relative growth rate in the presence of the two furan aldehydes, i.e. furfural and HMF (Fig. 1). The relative growth rate of the deletion mutant of *RPN4*, which is an important transcription factor for homeostatic regulation of the proteasome, was < 0.5 when cultivated with coniferyl aldehyde and spruce pretreatment liquid.

*PDR1*, *PDR3*, *PDR8*, *RDR1*, *YRR1* and *YRM1* are TFs specifically involved in MDR. Among these six deletion mutants, only the *PDR3* deletion mutant had a relative growth rate which was > 1.1 when cultivated with the pretreatment liquids (Fig. 1). The relative growth rates of the other five deletion mutants in this group were < 0.9 in the cultivations with inhibitors and pretreatment liquids.

*ADR1*, *CAT8*, *GAL4* and *MIG1* are four genes involved in carbon source response. The relative growth rates of the *CAT8* and *GAL4* mutants were < 0.9 in the cultivations with inhibitors and pretreatment liquids (Fig. 1).

The results suggested that the deletion mutant of *LEU3* had increased resistance to coniferyl aldehyde, bagasse pretreatment liquid, and spruce pretreatment liquid, but might be more sensitive than the control to furfural and HMF (Fig. 1). The relative growth rate of the deletion mutant of *PUT3*, another transcription factor involved in regulation of amino acid biosynthesis, was < 0.9 in cultivations with inhibitors and pretreatment liquids.

*DAL81* and *GZF3* are involved in the regulation of nitrogen catabolism. Similarly to that of the *LEU3* deletion mutant, the relative growth rates of the deletion mutant of *DAL81* were > 1.1 with coniferyl aldehyde and pretreatment liquids, but < 0.9 with furfural and HMF (Fig. 1). The *GZF3* deletion mutant was sensitive to all inhibitors and pretreatment liquids (relative growth rates < 0.9).

The relative growth rates of the deletion mutants of *ECM22* and *UPC2*, which are TFs involved in the regulation of sterol biosynthesis, were < 0.9 when cultivated with furan aldehydes and pretreatment liquids, but were in between 0.9 and 1.1 when cultivated with coniferyl aldehyde (Fig. 1).

### Microtitre plate experiment with transformants

**Fig. 2:**
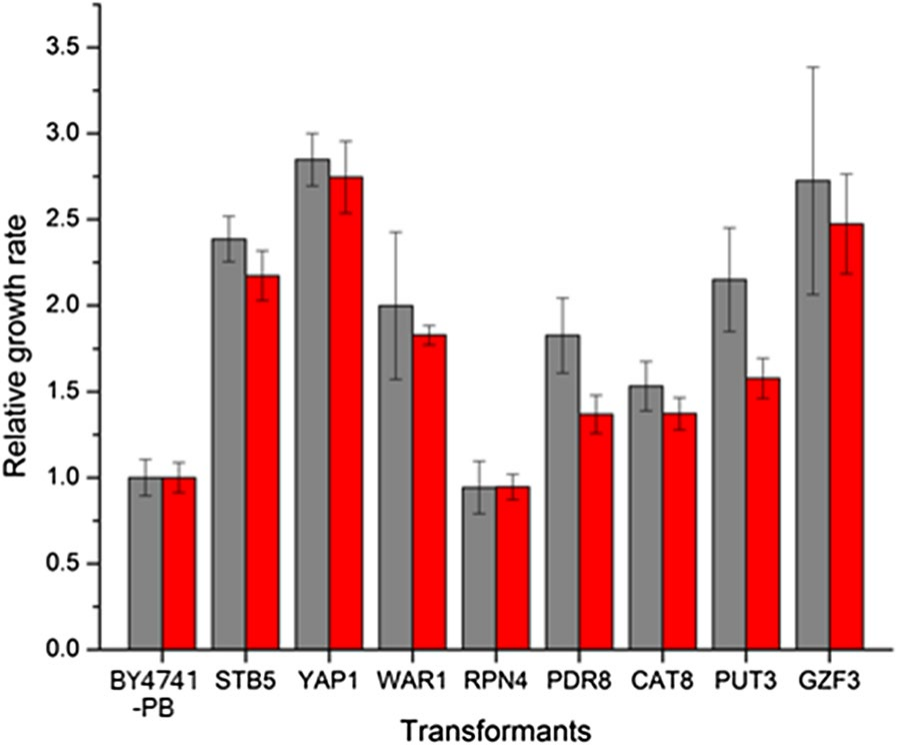
Relative growth rate of the transformants. The transformants were cultivated with sugarcane bagasse pretreatment liquid (grey square) and spruce pretreatment liquid (red square)

Eight of the TFs (*STB5*, *YAP1*, *WAR1*, *RPN4*, *CAT*8, *PDR8*, *PUT3* and *GZF3*), whose deletion mutants had relative growth rates under 0.7 (very sensitive) with both the sugarcane bagasse and the spruce pretreatment liquids (Additional file 1: Table S1), were overexpressed in *S. cerevisiae* BY4741 under the control of the potent *PGK1* promoter. The tolerance of the transformants to the sugarcane bagasse pretreatment liquid and the spruce pretreatment liquid were examined with microtiter plates. Except for the transformants of *RPN4*, all the transformants showed enhanced tolerance to the pretreatment liquids compared with the control transformants (Fig. 2). The relative growth rate of the transformants of *YAP1* were the highest among all the transformants, and were 2.848 ± 0.153 and 2.746 ± 0.209 with the bagasse pretreatment liquid and the spruce pretreatment liquid, respectively. The transformants of *STB5*, the other transcription factor directly related to oxidative stress, also had relative growth rates > 2.0 with the two pretreatment liquids. The overexpression of the transcription factors from other categories, such as *WAR1* (acid stress adaption), *PDR8* (pleiotropic drug resistance), *CAT1* (carbon source responsive), *PUT3* (amino-acid biosynthesis) and *GZF3* (nitrogen catabolism), were found to enhance the tolerance of the yeast strains to the two pretreatment liquids (Fig. 2).

The relative growth rate of the deletion mutant of *RPN4* was < 0.9 when cultivated with the two pretreatment liquids. With the spruce pretreatment liquid, the relative growth rate was even < 0.5. However, the overexpression of *RPN4* did not improve the tolerance of the transformants to the same pretreatment liquids.

### Microtitre-plate experiments and flask experiments with permutations of deletion and overexpression of *STB5* and *YAP1*

Deletion mutations of *STB5* and *YAP1*, the two TFs related to oxidative stress adaption, were consistently more sensitive to specific inhibitors and to pretreatment liquids. Further experiments were carried out to study their relationship with regard to inhibitor resistance and to see if it was possible to distinguish differences that the deletion mutant screening experiment (Fig. 1) did not reveal. *STB5* was overexpressed in the deletion mutant of *YAP1* (transformant denoted SIY), and *YAP1* was overexpressed in the deletion mutant of *STB5* (transformant denoted YIS). In addition, the deletion mutants of *YAP1* (M-Y) and *STB5* (M-S), the control strain for mutants (BY4741), the control for transformants (BY4741-PB), and the transformants overexpressing *YAP1* (T-Y) and *STB5* (T-S) were included in the experiment. The cell growth (OD_620_) of the deletion mutants and transformants was measured after 30 and 156 h of incubation (Table 1).

**Table 1:** Cell growth of STB5 and YAP1 deletion mutants and transformants in the microtiter plate assay

Strains, mutants and transformants^a,b^	Cell growth (OD_620_)									
	Coniferyl aldehyde (1.2 mM)		Furfural (17.0 mM)		HMF (24.0 mM)		BPL		SPL	
	30 h	156 h	30 h	156 h	30 h	156 h	30 h	156 h	30 h	156 h
BY4741	1.06 ± 0.02	1.24 ± 0.02	1.12 ± 0.02	1.14 ± 0.02	0.82 ± 0.02	1.04 ± 0.02	0.56 ± 0.02	1.07 ± 0.02	0.60 ± 0.01	1.10 ± 0.01
BY4741-PB	0.37 ± 0.15	1.41 ± 0.02	0.73 ± 0.05	1.10 ± 0.02	0.54 ± 0.04	0.96 ± 0.05	0.26 ± 0.09	1.18 ± 0.03	0.61 ± 0.06	1.33 ± 0.01
M-Y	0.08 ± 0.01	0.04 ± 0.01	0.79 ± 0.02	1.11 ± 0.01	0.59 ± 0.02	0.90 ± 0.01	0.11 ± 0.01	1.02 ± 0.02	0.12 ± 0.01	1.37 ± 0.03
M-S	0.06 ± 0.01	0.68 ± 0.12	0.06 ± 0.01	1.10 ± 0.05	0.09 ± 0.01	0.16 ± 0.02	0.06 ± 0.01	0.75 ± 0.05	0.08 ± 0.01	1.01 ± 0.03
SIY	0.08 ± 0.01	0.05 ± 0.01	0.66 ± 0.02	1.13 ± 0.02	0.50 ± 0.02	0.86 ± 0.02	0.10 ± 0.01	0.96 ± 0.08	0.12 ± 0.01	1.29 ± 0.02
YIS	0.08 ± 0.01	1.14 ± 0.03	0.08 ± 0.01	1.02 ± 0.01	0.18 ± 0.01	0.67 ± 0.02	0.10 ± 0.02	0.81 ± 0.01	0.20 ± 0.01	0.82 ± 0.04
T-S	0.80 ± 0.13	1.28 ± 0.05	0.96 ± 0.04	1.16 ± 0.01	0.70 ± 0.05	1.01 ± 0.02	0.71 ± 0.04	1.00 ± 0.04	0.76 ± 0.04	1.16 ± 0.02
T-Y	0.92 ± 0.07	1.13 ± 0.06	0.67 ± 0.06	1.05 ± 0.02	0.93 ± 0.04	1.12 ± 0.07	0.73 ± 0.05	0.89 ± 0.03	0.82 ± 0.02	1.22 ± 0.04

^a^BY4741, BY4741 host strain; BY4741-PB, BY4741 control transformant with plasmid backbone (PB) without insert; M-Y, deletion mutant of YAP1; M-S, deletion mutant of STB5; SIY, STB5 overexpressed in deletion mutant of YAP1; YIS, YAP1 overexpressed in deletion mutant of STB5; T-S, BY4741 transformant of STB5; T-Y, BY4741 transformant of YAP1

^b^The values were corrected as described in “Methods” section

After 30 h of incubation, the control transformant BY4741-PB did not exhibit more resistance than the host strain BY4741. Thus, as expected, transformation with the plasmid backbone did not lead to elevated resistance to inhibitors. M-Y and M-S were more sensitive than the BY4741 control to coniferyl aldehyde, furfural, HMF and to the two pretreatment liquids after 30 h incubation. After 156 h incubation, M-Y did not grow in coniferyl aldehyde, and was more sensitive than M-S, which grew to the relatively high OD_620_ of 0.68 (Table 1). After 156 h of incubation in medium with HMF, M-S grew to an (OD_620_) of only 0.16, and was more sensitive than M-Y, which grew to 0.90. This indicated that *STB5* was more important than *YAP1* for the adaptation of yeast to HMF. After 30 h incubation, M-S was more sensitive to furfural than M-Y, but the two deletion mutants grew to a similar level after 156 h incubation. The sensitivity of SIY to the inhibitors and the pretreatment liquids were similar to that of M-Y. Neither M-Y nor SIY grew with 1.1 mM coniferyl aldehyde after 156 h incubation, indicating that *YAP1* is vital for the resistance of yeast to coniferyl aldehyde, and it was not possible to substitute *STB5* for *YAP1* with regard to coniferyl aldehyde resistance. After 30 h incubation with inhibitors and pretreatment liquids, YIS grew poorer than BY4741 and BY4741-PB, but better than M-S (Table 1). That indicated that *YAP1* could only partially compensate for STB5 with regard to resistance against the inhibitors and the pretreatment liquids. After 156 h incubation with coniferyl aldehyde and HMF, the growth differences between YIS and M-S became larger. With coniferyl aldehyde, YIS grew to OD_620_ 1.14, while M-S grew to 0.68. With HMF, YIS grew to OD_620_ 0.67, while M-S grew to only 0.16. The result indicated that overexpression of *YAP1* in M-S could partially relieve the sensitivity of M-S to coniferyl aldehyde and HMF, and that *YAP1* partially compensated for *STB5* with regard to resistance against the two inhibitors.

After 30 h incubation, T-S and T-Y were more resistant than BY4741-PB in most of the cases (except for that T-Y was slightly more sensitive than BY4741-PB to furfural) (Table 1). Comparing T-S and SIY, T-S was more resistant than SIY after 30 h incubation with all the inhibitors and pretreatment liquids. T-Y was also more resistant than YIS in all the cases after 30 h incubation. The results indicated that even though either of *YAP1* or *STB5* was overexpressed, the deletion of the other TF of those two TFs would impair the resistance of yeast to the inhibitors and pretreatment liquids. That meant that either *YAP1* or *STB5* was indispensable for good resistance.

**Fig. 3:**
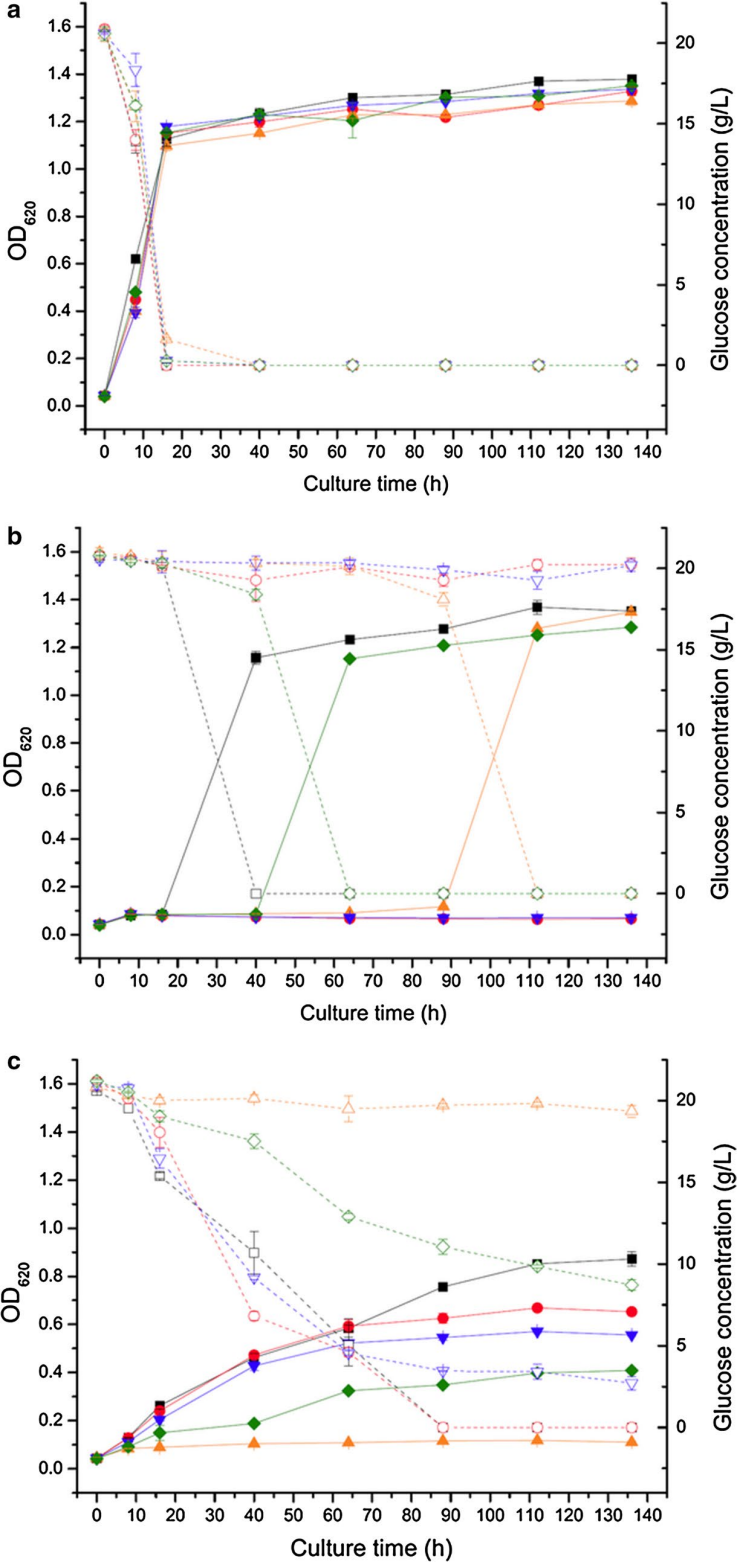
Cell growth and glucose consumption during flask experiments. BY4741, M-Y, M-S, SIY and YIS were cultivated in control medium (**a**), medium with 1.1 mM coniferyl aldehyde (**b**), and medium with 24 mM HMF (**c**). The data indicate: OD of BY4741 (filled black square), M-Y (filled red circle), M-S (filled orange triangle), SIY (filled inverted blue triangle), YIS (filled green rhombus), and glucose concentration of BY4741 (open black square), M-Y (open red circle), M-S (open orange triangle), SIY (open inverted blue triangle), YIS (open green rhombus)

BY4741, M-Y, M-S, SIY and YIS were selected for cultivation in flasks. Coniferyl aldehyde and HMF were used to further investigate the different involvements of *STB5* and *YAP1* in the resistance of yeast to the two compounds. Cell growth, glucose consumption, ethanol production and cell viability were measured during the fermentation. BY4741, the deletion mutants, and the transformants grew similarly in culture medium without inhibitors (control medium). After 8 h cultivation, all mutants and transformants had entered the exponential phase, and after 16 h they had entered the stationary phase. Accordingly, after 16 h of cultivation the glucose in the control medium was below 2 g/l for all mutants and transformants (Fig. 3a). The resistance of the yeast cells to 1.1 mM coniferyl aldehyde was in an order BY4741 > YIS > M-S > M-Y = SIY (Fig. 3b). All five mutants and transformants were still in the lag phase after 16 h cultivation (Fig. 3b). BY4741 started to grow between 16 and 32 h, and reached the stationary phase before 32 h. In accordance with this, the glucose was almost depleted by BY4741 between 16 and 32 h (Fig. 3b). YIS started to grow after 32 h cultivation, and reached the stationary phase before 64 h. In agreement with that, YIS consumed almost all the glucose between 32 and 64 h (Fig. 3b). M-S entered the exponential phase after 88 h cultivation, and reached the stationary phase before 112 h. The glucose was accordingly consumed during this period. M-Y and SIY could not grow with 1.1 mM coniferyl aldehyde after 136 h incubation, and the glucose was not consumed by M-Y and SIY (Fig. 3b). The resistance of the yeast cells to 24.0 mM HMF was in an order BY4741 > M-Y > SIY > YIS > M-S (Fig. 3c). BY4741 and M-Y grew similarly until 64 h cultivation, and both reached an OD of around 0.6 at 64 h (Fig. 3c). However, BY4741 grew faster than M-Y after 64 h, and reached OD 0.87 at 136 h, while M-Y reached OD 0.65 at the same time. The glucose in culture medium with HMF was consumed before 88 h by both BY4741 and M-Y. SIY grew slower than M-Y. The OD of the SIY culture was 0.52 at 64 h, and 0.56 at 136 h. The glucose concentration of SIY in medium with HMF was 4.56 g/l at 64 h, and 2.72 g/l glucose was left at 136 h. YIS grew slower than SIY. The OD of YIS reached 0.41 after 136 h cultivation, and 8.71 g/l glucose was left in the culture medium at 136 h. Even after 136 h of cultivation, M-S did not grow with 24.0 mM HMF, and, accordingly, almost no glucose was consumed (Fig. 3c).

With the control medium, the volumetric ethanol productivity (*Q*_*16h*_) of the five mutants and transformants reached about 0.55 g/l/h (Table 2). With coniferyl aldehyde in the culture medium, none of the five mutants and transformants produced any ethanol before 16 h. After 136 h fermentation with coniferyl aldehyde, the ethanol yields (*Y*_*E/G*_) of BY4741, M-S and YIS were 0.349, 0.375, and 0.317 g/g, respectively, while the ethanol yields of the M-Y and the SIY cultures were not even detectable. When cultivated with HMF, *Q*_*16h*_ of BY4741, M-Y and SIY were about 0.1 g/l/h, which was much higher than that of YIS. M-S did not produce any ethanol in the 136 h fermentation with HMF.

**Table 2:** Ethanol productivity and ethanol yield of STB5 and YAP1 deletion mutants and transformants in flask cultivations

Strains, mutants and transformants^a^	Inhibitor	*Q*_*16h*_ (g/l/h)^b^	*q*_*16h*_ (g/g/h)^b^	*Y*_*E/G*_ (g/g)^c^
BY4741	N/A^d^	0.58 ± 0.01	3.93 ± 0.04	0.463 ± 0.011
	Coniferyl aldehyde	NDTD	NDTD	0.349 ± 0.014
	HMF	0.08 ± 0.03	0.58 ± 0.15	0.222 ± 0.007
M-Y	N/A	0.55 ± 0.04	3.85 ± 0.16	0.437 ± 0.031
	Coniferyl aldehyde	NDTD	NDTD	NDTD
	HMF	0.12 ± 0.02	0.81 ± 0.09	0.182 ± 0.007
M-S	N/A	0.52 ± 0.03	3.62 ± 0.08	0.413 ± 0.021
	Coniferyl aldehyde	NDTD	NDTD	0.375 ± 0.023
	HMF	NDTD	NDTD	NDTD
SIY	N/A	0.54 ± 0.02	3.67 ± 0.08	0.434 ± 0.020
	Coniferyl aldehyde	NDTD	NDTD	NDTD
	HMF	0.11 ± 0.01	0.74 ± 0.01	0.241 ± 0.005
YIS	N/A	0.55 ± 0.01	3.91 ± 0.03	0.440 ± 0.005
	Coniferyl aldehyde	NDTD	NDTD	0.317 ± 0.005
	HMF	0.04 ± 0.01	0.28 ± 0.01	0.115 ± 0.023

*NDTD* none detected

^a^BY4741, BY4741 host strain; M-Y, deletion mutant of YAP1; M-S, deletion mutant of STB5; SIY, STB5 overexpressed in deletion mutant of YAP1; YIS, YAP1 overexpressed in deletion mutant of STB5

^b^Calculations based on the results within the first 16 h of fermentation. *Q* is the volumetric ethanol productivity, and *q* is the specific ethanol productivity

^c^ *Y*_*E/G*_ is the ethanol yield on the initial amount of fermentable sugar (glucose). The calculations are based on the maximum ethanol concentrations obtained within 136 h of fermentation

^d^N/A not applicable; no inhibitor was added to the culture medium

The cell viability was measured after 8 h of cultivation with coniferyl aldehyde, HMF and the control medium. In all the cultures, less than 10% of the cells were dead and had no intact cell membranes. However, the portion of cells with intact cell membrane was lower in cultures with coniferyl aldehyde than in cultures with control medium, especially with regard to M-Y.

## Discussion

The engineering of microbial strains has been an important technique for production of biofuels and bioproducts [14–16]. Knowledge of resistance of microorganisms to stress conditions is important for engineering robust microbial strains. Besides the development of novel carbohydrate-utilization pathways and the overexpression of cellulase in *S. cerevisiae* [17], development of robust yeast strains with greater resistance to fermentation inhibitors is important for efficient conversion of lignocellulosic feedstocks to cellulosic ethanol and other commodities [18]. A better understanding of the regulation of the resistance of yeast to lignocellulose-derived inhibitors, as provided by this investigation, facilitates characterization and engineering of hyper-resistant strains.

Using deletion mutants we screened the involvement of 29 MDR-related TFs with respect to resistance to three model inhibitors, coniferyl aldehyde, furfural, and HMF, and two pretreatment liquids, one from sugarcane bagasse and the other from spruce. All deletion mutants investigated showed increased sensitivity or increased resistance to at least one compound or pretreatment liquid, indicating that all those MDR-related TFs in some way were involved in the resistance to lignocellulose-derived inhibitors.

As 13 out of 29 TFs gave the same response for all the three model inhibitors as for the two pretreatment liquids, the set of model inhibitors chosen was well connected with the inhibitory effects of complex lignocellulosic hydrolysates. However, the relative growth rate of the deletion mutant of *CAD1* was < 0.9 when cultivated with the pretreatment liquids, but between 0.9 and 1.1 (not sensitive) when cultivated with the three specific inhibitors. The relative growth rate of the deletion mutants of *HAA1* was > 1.1 when cultivated with the pretreatment liquids, but between 0.9 and 1.1 (not resistant) when cultivated with the three specific inhibitors. The results therefore also indicate that the three model inhibitors do not cover all inhibitory effects of the pretreatment liquids, which is expected as there are many other inhibitors that can affect yeast cell growth [1, 19].

Using comparative transcriptome analysis, *YAP1*, *PDR1*, *PDR3*, *RPN4*, and *HSF1* were proposed to be key TFs of yeast in response to stress induced by HMF [20]. The TFs included in our study cover four of the five proposed key TFs for HMF resistance, and the results show that it was none of them but instead *STB5* that was most important with regard to HMF resistance. The discrepancy is probably due to the different approaches taken. We evaluated the involvement of the TFs in the response to the inhibitors through the relative growth rates of deletion mutants and transformants, not through transcriptomics as Ma and Liu [20]. The approach taken in our study seems more advantageous for finding proteins that are truly important for resistance, as products of genes that are not much induced in microarray analysis studies (e.g. *STB5*) might be very important for the adaption to the inhibitor. Furthermore, transcripts differ in stability and their abundance may not directly reflect the abundance of the corresponding proteins. The effect of different TFs on transcription levels may also differ.

The *RPN4* transcription factor stimulates expression of proteasome genes, and is rapidly degraded by the 26S proteasome [21]. Disruption of the Rpn4-induced proteasome expression in *S. cerevisiae* reduces cell viability under stressed conditions, and proteasomal degradation of Rpn4p in *S. cerevisiae* is critical for cell viability under stressed conditions [21]. In agreement with that, our result indicates that either the deletion or the over-expression of *RPN4* with a potent promoter (*PGK1*) of a multicopy plasmid (pAJ401) was not good for the resistance of the cells to the pretreatment liquids.

In order to predict more genes which might be important for the resistance to the pretreatment liquids, the genes regulated by the seven transcription factors (*STB5*, *YAP1*, *WAR1*, *PDR8*, *CAT8*, *PUT3* and *GZF3*), whose overexpression enhanced the tolerance of the yeast cells to the pretreatment liquids, were analyzed with MIPS Functional Categories using a p value cut-off of 0.01 for significance in the MIPS functional classification analysis [22] (Additional file 2: Table S2). The analysis showed that genes belonging to two functional categories (as defined by MIPS Functional Categories), C-compound and carbohydrate transport [20.01.03] and detoxification [32.07], are under regulation of three of those seven transcription factors (Additional file 2: Table S2). Genes in those two functional categories are predicted to be potentially important for the resistance of yeast to the fermentation inhibitors in the pretreatment liquids, and could be further studied with regard to engineering the resistance of yeast to lignocellulose-derived inhibitors.

The deletion mutants of *STB5* and *YAP1*, the two TFs involved in oxidative stress, were consistently sensitive to all model inhibitors and pretreatment liquids. Among the 29 deletion mutants that were compared, the deletion mutant of *STB5* was most sensitive to the furan aldehydes (the relative growth rates in media with furfural and HMF were 0.135 and 0.188, respectively), while the deletion mutant of *YAP1* was most sensitive to coniferyl aldehyde (growth not detectable) (Additional file 1: Table S1). This result indicates that adaptation of yeast cells to oxidative stress is critical for resistance to lignocellulose-derived inhibitors. *STB5* encodes a zinc transcription factor protein, which is required as a basal regulator of the PPP (pentose phosphate pathway) in *S. cerevisiae* [23]. Yeast produces NADPH through the PPP, and NADPH has been found to be important for the resistance of yeast to HMF [24] and furfural [25]. *YAP1* is the major oxidative stress transcription factor in *S. cerevisiae* [26]. It is involved in stress response, which offers protection against a variety of different forms of stress potentially induced by aldehyde inhibitors through damage of the cell membrane, cell wall and DNA and RNA synthesis [9]. Moreover, both furfural and HMF deplete cellular glutathione levels and accumulate reactive oxygen species [27]. *YAP1* is involved in the glutathione pathway, and is important in the detoxification of intracellular reactive oxygen species (ROS) [26]. The fermentability of the hydrolysates could be improved dramatically by treatment with reducing agents, such as dithionite and sulfite [28], which could react with aromatic compounds and furan aldehydes in the hydrolysates [29]. The fermentability of hydrolysates can also be improved by oxidation catalyzed by phenol-oxidizing enzymes such as laccases and peroxidases [30]. These findings indicate that the oxidation–reduction states of hydrolysates are relevant with regard to their toxicity. With that as background it makes sense that *STB5* and *YAP1*, the two TFs involved in oxidative stress, are important for the resistance of yeast to lignocellulose-derived inhibitors.

*HAA1* and *WAR1* are both involved in acid stress adaption [31], but differed considerably in the screening experiments with the deletion mutants. HAA1 is the main regulator for the adaption of yeast cells to acetic acid [31]. WAR1 is the regulator of PDR12, a plasma membrane protein which confers resistance of yeast to lipophilic organic acids [32]. High concentrations of aliphatic carboxylic acids, such as acetic acid, formic acid, and levulinic acid, and aromatic acids are well known to inhibit fermentations [1, 33].

The deletion mutants of *CIN5* and *MSN2*, which have been found to be involved in osmotic stress [34, 35], gave the same pattern, as the furans always gave higher values of relative growth rate than the other inhibitors (Fig. 1). According to YEASTRACT [36], *CIN5* and *MSN2* co-regulate 83 genes (through DNA binding and expression evidence). The screening results indicate that these two TFs have a similar function in the resistance to lignocellulose-derived inhibitors.

Six of the TFs investigated are specifically related to multidrug/pleiotropic drug resistance (Fig. 1). *PDR1* is involved in the resistance to multiple drugs of unrelated structure and function, such as cycloheximide, oligomycin, and venturicidin [37]. Disruption of both *PDR1* and *PDR3* resulted in high drug sensitivity to cycloheximide, oligomycin, and chloramphenicol, whereas disruption of only *PDR3* had a limited or undetectable effect [38]. Even though *PDR1* and *PRD3* are homologs to each other [38], the deletion mutants of these two TFs behaved oppositely with regard to the resistance to the pretreatment liquids. *PDR1* and *PDR3* have complex interactions exerting their regulatory functions, and *PDR3* can partially suppress positive regulation of Pdr1p [39]. That can explain why the deletion mutant of *PDR1* was sensitive to all the inhibitory substances tested in this study, while the deletion mutant of *PDR3* was not. *YRR1* is required for resistance to 4-nitroquinoline *N*-oxide [40]. Our results showed that *YRR1* was also required for resistance to the lignocellulose-derived inhibitors and the pretreatment liquids. *YRR1* and *YRM1* are paralogs involved in MDR, and *YRM1* has been found to partially mimic the effects of *YRR1* in its absence [41]. This agrees well with our finding that the deletion mutants of *YRR1* and *YRM1* were sensitive to all the three inhibitors and to the two pretreatment liquids.

Among the TFs involved in the regulation of metabolism, including carbon source responsive, amino acid biosynthesis and nitrogen catabolism, *LEU3* and *DAL81* behaved similarly. *LEU3* participates in the transcriptional regulation of the branched-chain amino-acid biosynthetic pathways [42]. *DAL81* is a positive regulator of genes in multiple nitrogen degradation pathways [43]. In accordance with expression evidence compiled in YEASTRACT [36], *LEU3* regulates the expression of *DAL81*. Our results indicate that amino-acid biosynthesis and nitrogen degradation pathways are of importance for yeast in the resistance to the pretreatment liquids.

*ECM22* and *UPC2* are involved in the regulation of the cell-membrane composition of yeast [44]. The relative growth rates of the deletion mutants of both of these TFs were < 0.9 when cultivated in media with furfural, HMF and pretreatment liquids, but were in between 0.9 and 1.1 (not sensitive) when cultivated in medium with coniferyl aldehyde. Complete sterol biosynthesis in yeast requires NADH/NADPH [45], and may be impaired by the presence of furan aldehydes, the reduction of which require NADH/NADPH. The deletion of *ECM22* and *UPC2* might aggravate adverse effects on the sterol biosynthesis when the cells were cultivated with furan aldehydes. Even though phenolics have been found to interfere with the cell membrane [46], the deletion mutants of *ECM22* and *UPC2* were not sensitive to coniferyl aldehyde. That may because the concentration of coniferyl aldehyde in the experiments (1.0 mM) was much lower than that of furfural (10.4 mM) and HMF (14.0 mM).

The experiments with permutations of deletion and overexpression of *STB5* and *YAP1* indicated that the roles of the two TFs were complementary with regard to HMF resistance, in a sense that *STB5* and *YAP1* could at least in part take over the role of each other when the other TF was lacking, but distinct with regard to coniferyl aldehyde resistance, in a sense that *STB5* could not take over the role of *YAP1* in the resistance to coniferyl aldehyde when the latter TF was lacking. Stb5p activates most of the genes of the PPP (*ZWF1*, *SOL3*, *GND1*, *GND2*, *TKL1*, *TAL1* and *RKI1*) [23]. Several of those genes (*GND1*, *TAL1*, *ZWF1*, *RKI1* and *TKL1*) are also regulated by Yap1p. Interestingly, three proteins localized in the outer membrane of the mitochondrion, Uth1p, Por1p and Nde1p, belong to the *STB5* regulon but not to the *YAP1* regulon (data from YEASTRACT). They are required for the maintenance of mitochondrial functions, and some of them have been found to be involved in oxidative stress response [47–49]. The extended role played by Stb5p in the regulation of the PPP compared to that of Yap1p can explain why Stb5p was more important than Yap1p with respect to resistance against furan aldehydes. Flr1p and Atr1p, two MDR proteins that have been found to be involved in the resistance to coniferyl aldehyde [6, 7], are positively regulated by Yap1p, but not by Stb5p. That agrees with the result that overexpression of *STB5* cannot compensate with regard to the resistance to coniferyl aldehyde when *YAP1* is deleted. Furthermore, the main metabolic enzymes in the glycolysis and pyruvate–ethanol pathways, including Cdc19p, Pgk1p, Dld3p, Tdh3p, Eno2p, Tdh1p, Tdh2p, Fba1p, Pdc1p and Adh1p, are positively regulated by Yap1p, but not by Stb5p (YEASTRACT). Many heat-shock and chaperone proteins (Ssa1p, Ssa2p, Ssb1p, Ssb2p, Hsp82p, Hsc82p and Sse1p) were also found to be up-regulated by only Yap1p. Those heat-shock and chaperone proteins and the proteins in the glycolytic pathway and the pyruvate-ethanol pathway may be involved in the resistance of yeast to coniferyl aldehyde.

Previous research has shown that coniferyl aldehyde is converted by yeast to coniferyl alcohol and dihydroconiferyl alcohol, and that the yeast cells started to grow only when the concentration of coniferyl aldehyde was decreased to a certain amount [33]. Our result agreed that the relatively low concentration of coniferyl aldehyde (1.1 mM) caused only a lag phase but did not cause lower final OD value. Strains overexpressing *STB5* have been found to not be able to grow on glucose [23]. Our results agree with that in the sense that the overexpression of *STB5* repressed the glucose consumption. However, in our experiments T-S and SIY could consume glucose partially.

Many inhibitors could inhibit not only the yeast cell growth but also the ethanol production [33]. Our results agree with that and showed that the ethanol yields of the samples from cultures with coniferyl aldehyde and HMF were lower than those from cultures with control medium. Coniferyl aldehyde caused a lag phase of the cell growth of yeast, which differed from the furan aldehydes which decreased the growth rate. The results indicate that the inhibitory mechanism of coniferyl aldehyde differs from those of the furan aldehydes.

The cell viability was measured after 8 h cultivation with coniferyl aldehyde, HMF and the control medium. Since the experiments were on purpose performed with concentrations of coniferyl aldehyde and HMF that were lower than would be lethal for the cultures, the majority of the cells of all mutants and transformants had intact membranes. However, the portion of cells with intact membrane was lower in cultures with coniferyl aldehyde than in cultures with control medium, especially for M-Y. Thus, the assay suggested that deletion of *YAP1* had a negative effect on cell viability, especially under stress conditions caused by coniferyl aldehyde.

## Conclusions

In this study, we have profiled a set of 29 deletion mutants of TFs related to MDR with regard to their roles in the resistance to toxic concentrations of lignocellulose-derived substances. All of the TFs studied were found to be involved in the resistance of yeast to coniferyl aldehyde, furfural, HMF, sugarcane bagasse pretreatment liquid or spruce pretreatment liquid. The overexpression of seven of the transcription factors improved the tolerance of yeast cells to the inhibitors in both sugarcane bagasse and spruce hydrolysates. Some deletion mutants (of e.g. *MSN2* and *LEU3*) seemed to be more resistant to the lignocellulose-derived inhibitors. Further experiments are needed to investigate the potential significance of this phenomenon. Moreover, the roles of *STB5* and *YAP1*, genes encoding TFs involved in oxidative stress response, were elucidated in detail with regard to resistance to coniferyl aldehyde and HMF. *STB5* was most important for yeast adaption to HMF, while *YAP1* was most important for the adaption to coniferyl aldehyde. The roles of the two TFs were complementary with regard to HMF resistance, but distinct with regard to coniferyl aldehyde resistance. The complementarity may be attributed to the roles played by the TFs encoded by *STB5* and *YAP1* in the regulation of the pentose phosphate pathway, while the distinct role played by the *YAP1* transcription factor in the resistance to coniferyl aldehyde may be attributed to its regulation of MDR proteins not affected by *STB5*. Our study clearly shows the important roles played by the MDR transcription factors in resistance to the multiple lignocellulose-derived inhibitors in hydrolysates. The transcription factors which are involved in the resistance of yeast to the inhibitors can be overexpressed to construct robust strain in biofuels and biochemicals production from lignocellulosic biomass. We predict that genes belonging to two functional categories (as defined by MIPS Functional Categories) are potentially important for the resistance of yeast to the inhibitors. Those genes can be further studied with regard to engineering the resistance of yeast. We also show the important role of *STB5* in the resistance to furan aldehydes, something that has been overlooked previously in studies based on transcriptomics. The information on the complementary and distinct roles of the transcription factors with regard to the resistance to lignocellulose-derived inhibitors is useful in future investigations on engineering hyper-resistant yeast for biomass conversion.

## Additional files

**Additional file 1: Table S1.** Relative growth rate of the deletion mutants of the transcription factors.

**Additional file 2: Table S2.** Number of genes regulated by the seven transcription factors, and associated MIPS Functional Categories.
