# Supplementary material for: Profiling of Saccharomyces cerevisiae transcription factors for engineering the resistance of yeast to lignocellulose-derived inhibitors in biomass conversion
Source: Microb Cell Fact. 2017 Nov 14;16:199. doi: 10.1186/s12934-017-0811-9 (PMC5686817; doi:10.1186/s12934-017-0811-9)
Supplement: Supplementary file 1 — Additional file 1: Table S1. Relative growth rate of the deletion mutants of the transcription factors. [file 12934_2017_811_MOESM1_ESM.doc]

**Table S1.** Relative growth rate of the deletion mutants of the transcription factors.^a^

| **Transcription factor** | **Classification** | **Relative growth rate of the deletion mutants**  **CFA Furfural HMF BPL SPL** | | | | |
| --- | --- | --- | --- | --- | --- | --- |
|  | **Oxidative stress** |  |  |  |  |  |
| STB5 | Zn(II)2Cys6 | 0.280±0.004 | 0.135±0.005 | 0.188±0.004 | 0.459±0.014 | 0.402±0.012 |
| YAP1 | bZIP | NTDT^b^ | 0.682±0.005 | 0.656±0.004 | 0.589±0.020 | 0.578±0.011 |
|  | **Acid stress** |  |  |  |  |  |
| HAA1 | Copper fist | 1.059±0.010 | 0.983±0.024 | 1.023±0.009 | 1.346±0.059 | 1.221±0.057 |
| WAR1 | Zn(II)2Cys6 | 0.646±0.014 | 0.544±0.034 | 0.574±0.011 | 0.616±0.039 | 0.545±0.006 |
|  | **Stress response** |  |  |  |  |  |
| ARR1 | bZIP | 1.156±0.029 | 0.952±0.028 | 0.975±0.022 | 0.705±0.066 | 0.951±0.024 |
| CAD1 | bZIP | 1.120±0.008 | 0.970±0.058 | 1.028±0.034 | 0.781±0.044 | 0.887±0.013 |
| CIN5 | bZIP | 1.060±0.007 | 2.075±0.040 | 1.671±0.021 | 1.307±0.033 | 1.216±0.019 |
| CRZ1 | C2H2 ZF | 1.280±0.144 | 1.039±0.071 | 1.030±0.085 | 1.111±0.027 | 1.151±0.076 |
| MSN2 | Cys2His2 | 1.101±0.016 | 1.816±0.064 | 1.705±0.022 | 1.221±0.019 | 1.203±0.024 |
| MSN4 | Cys2His2 | 1.088±0.033 | 0.788±0.055 | 0.787±0.027 | 0.658±0.011 | 0.754±0.020 |
| RPN4 | C2H2 ZF | 0.424±0.014 | 0.822±0.035 | 0.916±0.029 | 0.576±0.054 | 0.479±0.020 |
| SMP1 | The MADS-box family | 1.237±0.068 | 1.032±0.043 | 1.016±0.027 | 0.840±0.016 | 0.958±0.039 |
| YAP7 | bZIP | 1.216±0.050 | 1.022±0.019 | 1.014±0.014 | 0.771±0.007 | 0.944±0.028 |
|  | **Multidrug/ pleiotropic drug resistance** |  |  |  |  |  |
| PDR1 | Zn(II)2Cys6 | 0.710±0.025 | 0.750±0.020 | 0.834±0.017 | 0.804±0.013 | 0.857±0.014 |
| PDR3 | Zn(II)2Cys6 | 0.986±0.007 | 0.923±0.012 | 0.966±0.033 | 1.200±0.064 | 1.317±0.035 |
| PDR8 | Zn(II)2Cys6 | 0.428±0.023 | 0.564±0.015 | 0.594±0.010 | 0.507±0.014 | 0.546±0.012 |
| RDR1 | Zn(II)2Cys6 | 0.516±0.005 | 0.594±0.014 | 0.534±0.014 | 0.596±0.010 | 0.641±0.008 |
| YRR1 | Zn(II)2Cys6 | 0.471±0.019 | 0.705±0.014 | 0.579±0.020 | 0.805±0.021 | 0.715±0.021 |
| YRM1 | Zn(II)2Cys6 | 0.552±0.049 | 0.671±0.014 | 0.603±0.007 | 0.812±0.024 | 0.732±0.018 |
|  | **Carbon source**  **responsive** |  |  |  |  |  |
| ADR1 | Cys2His2 | 1.142±0.076 | 0.871±0.041 | 0.841±0.031 | 0.889±0.032 | 0.881±0.022 |
| CAT8 | Zn(II)2Cys6 | 0.897±0.048 | 0.728±0.039 | 0.784±0.017 | 0.621±0.010 | 0.611±0.016 |
| GAL4 | Zn(II)2Cys6 | 0.896±0.065 | 0.719±0.020 | 0.799±0.019 | 0.702±0.011 | 0.687±0.010 |
| MIG1 | Cys2His2 | 1.074±0.033 | 1.064±0.017 | 1.206±0.010 | 0.623±0.013 | 0.981±0.016 |
|  | **Amino acid biosynthesis** |  |  |  |  |  |
| LEU3 | Zn(II)2Cys6 | 1.856±0.219 | 0.846±0.021 | 0.733±0.053 | 2.259±0.089 | 1.951±0.120 |
| PUT3 | Zn(II)2Cys6 | 0.880±0.023 | 0.761±0.024 | 0.767±0.028 | 0.581±0.013 | 0.684±0.018 |
|  | **Nitrogen catabolism** |  |  |  |  |  |
| DAL81 | Zn(II)2Cys6 | 1.142±0.022 | 0.620±0.022 | 0.532±0.005 | 1.372±0.042 | 1.559±0.082 |
| GZF3 | GATA zinc finger protein | 0.691±0.057 | 0.513±0.025 | 0.520±0.016 | 0.585±0.003 | 0.643±0.023 |
|  | **Cell membrane composition** |  |  |  |  |  |
| ECM22 | Zn(II)2Cys6 | 1.001±0.056 | 0.841±0.043 | 0.885±0.029 | 0.691±0.024 | 0.794±0.020 |
| UPC2 | Zn(II)2Cys6 | 0.936±0.061 | 0.816±0.040 | 0.887±0.033 | 0.609±0.035 | 0.756±0.015 |

^a^The result with the NGG1 deletion mutant was not included in the table, since the growth of the NGG1 deletion mutant in the SC medium was slower than that of BY4741.

^b^NTDT, Not detected; the YAP1 deletion mutant did not grow with 1.0 mM coniferyl aldehyde in the medium.
