# Supplementary material for: Profiling of Saccharomyces cerevisiae transcription factors for engineering the resistance of yeast to lignocellulose-derived inhibitors in biomass conversion
Source: Microb Cell Fact. 2017 Nov 14;16:199. doi: 10.1186/s12934-017-0811-9 (PMC5686817; doi:10.1186/s12934-017-0811-9)
Supplement: Supplementary file 2 — Additional file 2: Table S2. Number of genes regulated by the seven transcription factors, and associated MIPS Functional Categories. [file 12934_2017_811_MOESM2_ESM.docx]

**Table S2.** Number of genes regulated by the seven transcription factors, and associated MIPS Functional Categories.^a, b, c, d^

| **Category** | | **k_STB5_** | **k_YAP1_** | **k_WAR1_** | **k_PDR8_** | **k_CAT8_** | **k_PUT3_** | **k_GZF3_** | **f** |
| --- | --- | --- | --- | --- | --- | --- | --- | --- | --- |
| METABOLISM | amino acid metabolism [01.01] |  | 20 |  |  |  |  |  | 39 |
|  | biosynthesis of glutamate [01.01.03.02.01] |  |  |  |  | 3 |  |  | 15 |
|  | degradation of proline [01.01.03.03.02] |  |  |  |  |  | 3 |  | 4 |
|  | biosynthesis of arginine [01.01.03.05.01] |  |  |  |  |  |  | 3 | 13 |
|  | metabolism of urea (urea cycle) [01.01.05.03] |  |  |  |  |  |  | 2 | 6 |
|  | metabolism of methionine [01.01.06.05] |  | 12 |  |  |  |  |  | 21 |
|  | biosynthesis of homocysteine [01.01.06.05.01.01] |  | 6 |  |  |  |  |  | 7 |
|  | aminoadipic acid pathway [01.01.06.06.01.03] |  |  |  |  |  |  | 3 | 9 |
|  | degradation of lysine [01.01.06.06.02] |  |  |  |  |  |  | 3 | 5 |
|  | biosynthesis of serine [01.01.09.02.01] |  | 6 |  |  |  |  |  | 7 |
|  | biosynthesis of cysteine [01.01.09.03.01] |  | 4 |  |  |  |  |  | 4 |
|  | metabolism of phenylalanine [01.01.09.04] |  | 11 |  |  |  |  |  | 13 |
|  | metabolism of tyrosine [01.01.09.05] |  | 11 |  |  |  |  |  | 14 |
|  | biosynthesis of valine [01.01.11.03.01] |  | 5 |  |  |  |  |  | 6 |
|  | biosynthesis of leucine [01.01.11.04.01] |  | 6 |  |  |  |  |  | 8 |
|  | nitrogen, sulfur and selenium metabolism [01.02] |  | 24 |  |  | 5 |  |  | 54 |
|  | sulfate assimilation [01.02.03.01] |  | 6 |  |  |  |  |  | 8 |
|  | regulation of nitrogen metabolism [01.02.07.01] |  |  |  |  |  |  | 3 | 8 |
|  | purine nucleotide/nucleoside/nucleobase anabolism [01.03.01.03] |  | 14 |  |  |  |  |  | 29 |
|  | C-compound and carbohydrate metabolism [01.05] |  | 96 |  |  | 12 |  |  | 223 |
|  | sugar, glucoside, polyol and carboxylate anabolism [01.05.02.04] |  |  | 2 |  |  |  |  | 35 |
|  | sugar, glucoside, polyol and carboxylate catabolism [01.05.02.07] |  | 39 |  |  | 6 |  |  | 81 |
|  | C-2 compound and organic acid catabolism [01.05.06.07] |  |  |  |  | 3 |  |  | 9 |
|  | metabolism of vitamins, cofactors, and prosthetic groups [01.07] |  | 19 |  |  |  |  |  | 43 |
|  | biosynthesis of vitamins, cofactors, and prosthetic groups [01.07.01] |  | 42 |  |  |  |  |  | 110 |
|  | metabolism of secondary products derived from glycine, L-serine and L-alanine [01.20.19] |  | 4 |  |  |  |  |  | 4 |
| ENERGY | glycolysis and gluconeogenesis [02.01] |  | 22 |  |  | 6 |  |  | 41 |
|  | glyoxylate cycle [02.04] |  |  |  |  | 4 |  |  | 9 |
|  | pentose-phosphate pathway [02.07] |  | 13 |  |  |  |  |  | 23 |
|  | tricarboxylic-acid pathway (citrate cycle, Krebs cycle, TCA cycle) [02.10] |  | 15 |  |  | 7 |  |  | 31 |
|  | respiration [02.13] |  | 26 |  |  |  |  |  | 59 |
|  | fermentation [02.16] |  | 16 |  |  |  |  |  | 24 |
|  | alcohol fermentation [02.16.01] |  | 9 |  |  |  |  |  | 13 |
|  | lactate fermentation [02.16.03] |  |  |  |  | 2 |  |  | 4 |
|  | metabolism of energy reserves (e.g. glycogen, trehalose) [02.19] |  | 24 |  | 2 |  |  |  | 56 |
| CELL CYCLE AND DNA PROCESSING | DNA topology [10.01.02] |  |  |  |  |  |  | 6 | 54 |
|  | somatic / mitotic recombination [10.01.05.03.03] |  |  |  |  |  |  | 5 | 21 |
| TRANSCRIPTION | rRNA synthesis [11.02.01] |  |  | 3 |  |  |  |  | 55 |
|  | rRNA processing [11.04.01] | 43 |  |  |  |  |  |  | 169 |
|  | rRNA modification [11.06.01] | 5 |  |  |  |  |  |  | 18 |
|  | ribosome biogenesis [12.01] | 16 |  |  |  |  |  |  | 64 |
|  | ribosomal proteins [12.01.01] |  | 144 |  |  |  |  |  | 246 |
|  | aminoacyl-tRNA-synthetases [12.10] |  | 18 |  |  |  |  |  | 39 |
| PROTEIN FATE | protein folding and stabilization [14.01] |  | 42 |  |  |  |  |  | 93 |
|  | protein processing (proteolytic) [14.07.11] |  | 27 |  |  |  |  |  | 63 |
|  | proteasomal degradation (ubiquitin/proteasomal pathway) [14.13.01.01] |  | 53 |  |  |  |  |  | 128 |
|  | RNA binding [16.03.03] | 23 |  |  |  |  |  |  | 189 |
|  | ATP binding [16.19.03] |  |  |  | 4 |  |  |  | 191 |
|  | NAD/NADP binding [16.21.07] |  | 19 |  |  |  |  |  | 36 |
|  | Fe/S binding [16.21.08] |  |  |  |  | 2 |  |  | 5 |
| REGULATION OF METABOLISM AND PROTEIN FUNCTION | regulator of transcription factor [18.02.09] |  |  |  |  |  |  | 4 | 37 |
| CELLULAR TRANSPORT, TRANSPORT FACILITIES AND TRANSPORT ROUTES | ion transport [20.01.01] |  |  |  |  |  | 2 |  | 7 |
|  | heavy metal ion transport (Cu+, Fe3+, etc.) [20.01.01.01.01] |  |  |  |  | 5 | 11 |  | 41 |
|  | siderophore-iron transport [20.01.01.01.01.01] |  |  |  |  |  | 10 |  | 12 |
|  | C-compound and carbohydrate transport [20.01.03]* |  |  | 2 |  | 6 |  | 5 | 34 |
|  | C4-dicarboxylate transport (e.g. malate, succinate, fumarate) [20.01.03.03] |  |  |  |  | 2 |  |  | 4 |
|  | amino acid/amino acid derivatives transport [20.01.07] |  |  |  |  | 6 |  |  | 45 |
|  | amine / polyamine transport [20.01.11] | 4 |  |  |  |  |  |  | 14 |
|  | electron transport [20.01.15] |  | 34 |  |  |  |  |  | 83 |
|  | drug/toxin transport [20.01.27] |  |  |  | 5 |  | 7 |  | 39 |
|  | transport facilities [20.03] |  |  |  |  | 7 |  |  | 87 |
|  | ion channels [20.03.01.01] | 4 |  |  |  |  |  |  | 10 |
|  | antiporter [20.03.02.03] |  |  |  |  | 3 |  |  | 7 |
|  | transport ATPases [20.03.22] |  |  |  | 2 |  |  |  | 53 |
|  | ABC transporters [20.03.25] |  |  |  | 4 |  |  |  | 28 |
|  | mitochondrial transport [20.09.04] |  |  |  |  | 10 |  |  | 104 |
|  | cellular import [20.09.18] |  | 37 |  |  | 7 |  |  | 90 |
| CELL RESCUE, DEFENSE AND VIRULENCE | stress response [32.01] |  | 75 |  |  |  |  |  | 162 |
|  | oxidative stress response [32.01.01] |  | 38 |  | 3 |  |  |  | 55 |
|  | heat shock response [32.01.05] |  | 11 |  |  |  |  | 3 | 20 |
|  | unfolded protein response (e.g. ER quality control) [32.01.07] |  | 36 |  |  |  |  |  | 69 |
|  | DNA damage response [32.01.09] |  |  |  |  |  |  | 6 | 77 |
|  | chemical agent resistance [32.05.01.03] | 5 |  |  | 4 |  |  |  | 22 |
|  | detoxification [32.07]* |  | 37 |  | 3 |  | 8 |  | 80 |
|  | detoxification by modification [32.07.03] |  | 8 |  |  |  |  |  | 8 |
|  | detoxification by export [32.07.05] |  |  |  | 1 |  |  |  | 3 |
|  | oxygen and radical detoxification [32.07.07] |  | 8 |  |  |  | 3 |  | 12 |
|  | catalase reaction [32.07.07.01] |  |  |  | 1 |  |  |  | 2 |
|  | peroxidase reaction [32.07.07.05] |  | 6 |  |  |  |  |  | 7 |
| INTERACTION WITH THE ENVIRONMENT | homeostasis of metal ions (Na, K, Ca etc.) [34.01.01.01] |  | 40 |  |  |  | 21 |  | 98 |
|  | osmosensing and response [34.11.03.13] | 6 |  |  |  |  |  |  | 35 |
|  | temperature perception and response [34.11.09] |  |  |  |  |  |  | 3 | 19 |
| CELL FATE | anti-apoptosis [40.10.02.01] | 2 |  |  |  |  |  |  | 2 |
| DEVELOPMENT | mating (fertilization) [41.01.01] |  |  |  |  |  |  | 7 | 69 |
| UNCLASSIFIED PROTEINS | UNCLASSIFIED PROTEINS [99] |  |  |  |  |  |  | 29 | 1378 |

^a^K_STB5_-k_GZF3_ indicate the number of genes of a specific category under the regulation of STB5- GZF3, respectively, and f is the number of genes of a specific category according to MIPS.

^b^Empty cells indicate that the number of genes was not significant with a p-value cutoff of 0.01 in the MIPS functional classification analysis.

^c^Functional Categories with genes which are under regulation of three transcription factors are marked with an asterisk (*).

^d^Data on genes regulated by the seven transcription factors were obtained from YEASTRACT, through documented direct and indirect evidence.
